# Evaluation of a flipped classroom approach to learning introductory epidemiology

**DOI:** 10.1186/s12909-018-1150-1

**Published:** 2018-04-02

**Authors:** Stephanie Shiau, Linda G. Kahn, Jonathan Platt, Chihua Li, Jason T. Guzman, Zachary G. Kornhauser, Katherine M. Keyes, Silvia S. Martins

**Affiliations:** 1 Department of Epidemiology, Columbia University Mailman School of Public Health, 722 West 168th Street, Room 509, New York, NY 10032 USA; 2 Department of Pediatrics, New York University School of Medicine, New York, NY USA; 3 Center for Teaching and Learning, Columbia University, New York, NY USA; 4 School of Professional Studies, Columbia University, New York, NY USA

**Keywords:** Public health, Flipped classroom, Epidemiology, Graduate level setting, Education, Instructional tecnology

## Abstract

**Background:**

Although the flipped classroom model has been widely adopted in medical education, reports on its use in graduate-level public health programs are limited. This study describes the design, implementation, and evaluation of a flipped classroom redesign of an introductory epidemiology course and compares it to a traditional model.

**Methods:**

One hundred fifty Masters-level students enrolled in an introductory epidemiology course with a traditional format (in-person lecture and discussion section, at-home assignment; 2015, *N* = 72) and a flipped classroom format (at-home lecture, in-person discussion section and assignment; 2016, *N* = 78). Using mixed methods, we compared student characteristics, examination scores, and end-of-course evaluations of the 2016 flipped classroom format and the 2015 traditional format. Data on the flipped classroom format, including pre- and post-course surveys, open-ended questions, self-reports of section leader teaching practices, and classroom observations, were evaluated.

**Results:**

There were no statistically significant differences in examination scores or students’ assessment of the course between 2015 (traditional) and 2016 (flipped). In 2016, 57.1% (36) of respondents to the end-of-course evaluation found watching video lectures at home to have a positive impact on their time management. Open-ended survey responses indicated a number of strengths of the flipped classroom approach, including the freedom to watch pre-recorded lectures at any time and the ability of section leaders to clarify targeted concepts. Suggestions for improvement focused on ways to increase regular interaction with lecturers.

**Conclusions:**

There was no significant difference in students’ performance on quantitative assessments comparing the traditional format to the flipped classroom format. The flipped format did allow for greater flexibility and applied learning opportunities at home and during discussion sections.

**Electronic supplementary material:**

The online version of this article (10.1186/s12909-018-1150-1) contains supplementary material, which is available to authorized users.

## Background

The “flipped” or “inverted” classroom is a pedagogical model first described by Lage, Platt, and Treglia in 2000 and later popularized by Bergmann and Sams in 2012 in which the traditional course components – an “in-person” classroom lecture and an “at home” assignment – are reversed [1–3]. Instead, in a flipped classroom, pre-recorded lectures are viewed outside of the classroom setting (at home), and in-person classroom time is devoted to interactive exercises, discussions, or group projects. This blended learning approach is intended to improve the efficacy of classroom learning by allowing students to control the timing and pace of their online learning and maximize their opportunity for active learning by engaging in class discussions and collaborative exercises in the company of peers and instructors [4, 5].

The use of flipped classroom approaches is growing in many health science fields, including nursing [6, 7], medical [8], dental education [9], pharmacy [10], and public health [11, 12]. A recent systematic review of nine studies of the use of the flipped classroom model in medical education concluded that while learning outcomes were the same or improved in flipped classes versus traditional lecture courses, students in the flipped classrooms consistently reported greater satisfaction [8]. A systematic review of the model’s use in graduate nursing education reached similar conclusions, while a meta-analysis of 11 randomized controlled trials in undergraduate nursing programs in China reported significantly higher theoretical knowledge and skill scores in flipped vs. traditional classrooms [8, 13]. To our knowledge, to date there has been only one other published report of flipping an introductory class in epidemiology, the basic science of public health [14]. In that case, conducted at the School of Public Health at the University of Saskatchewan, 80% of the students found the flipped classroom model to be somewhat or very effective, with higher ratings among international students compared to North American students. Final grades were comparable to those in the prior traditional format, but classroom effectiveness and satisfaction ratings were improved.

In Fall 2016, a group of master’s level public health students at Columbia University’s Mailman School of Public Health (MSPH) in New York City participated in a flipped classroom redesign of an introductory epidemiology course. This paper reports on the design, implementation, and evaluation of this course redesign. The aims of this project were to 1) implement a flipped classroom redesign of a master’s level introductory epidemiology course, 2) assess student’s self-reported learning gains throughout the course, 3) compare student course evaluations and examination results in the flipped model to the traditional model, and 4) analyze the advantages and disadvantages of the flipped classroom redesign.

## Methods

### Rationale for course redesign

Principles of Epidemiology is an introductory epidemiology course designed for students of public health taken by students enrolled in the Master of Science (MS) programs in Epidemiology, Biostatistics, and related programs. Approximately 75 students have enrolled in this course at the MSPH in the last 4 years. Over the past decade (2006–2016), different versions of the course have been offered, including a traditional format (3 in-person hours/week, including a 1.5-h lecture and 1.5-h discussion section), a digital format (1 to 1.5-h lectures, 1-h discussion sections, and open discussion boards offered only online), and an executive format (four in-person Friday-Sunday intensives including both lectures and discussion sections). In Fall 2014, we simultaneously ran both traditional and digital versions of Principles of Epidemiology for the first time; results revealed that these formats had different advantages and disadvantages. Students in the digital class enjoyed the flexibility of watching the videos of recorded lectures on their own time, but several noted that they would have preferred in-person discussion sections. Meanwhile, students in the traditional class (95.6%) overwhelmingly indicated that the discussion sections supported the learning goals of the course, but many—especially working students—commented that they found the three-hour evening back-to-back lecture-discussion section format difficult to manage. Discussion section leaders, doctoral students in the Department of Epidemiology, also observed that because traditional students did not have time to integrate the lecture material prior to the discussion sections that immediately followed, the effectiveness of the discussion sections was compromised. Other problems with the status quo included a lack of standardization across introductory epidemiology courses taught at MSPH, outdated materials, and a lack of opportunity for students to experience a real-world application of their knowledge. Having tested both a traditional version and digital version of Principles of Epidemiology, we chose to redesign the course according to a flipped classroom model that combined components of both. This was the first instance of a flipped classroom in the Department of Epidemiology at the MSPH.

### The course redesign

**Fig. 1:**
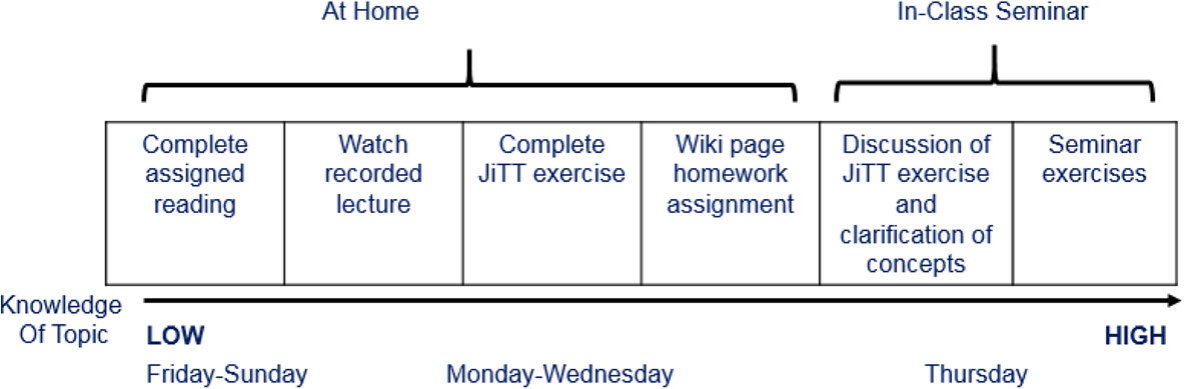
Diagram of flipped classroom schedule

We implemented a flipped classroom version of Principles of Epidemiology that incorporated the best aspects of the previous course iterations, such as the flexible online lectures and the active discussion sections, based on student and instructor evaluations. Course content was delivered through Canvas, an online education platform (Instructure, Salt Lake City, UT), and weekly in-person discussion sections with leaders. Figure 1 shows the flipped course format, designed for students to build their understanding of course concepts throughout each week using multiple modes of learning. Students were informed of the new approach through the syllabus (available online prior to registration), an introductory email from the professor at the beginning of the semester, and during the first in-person class session.

#### At home

Students were introduced to new material each week by completing assigned readings from textbooks and journal articles, then by watching recorded lectures given by faculty experts at MSPH on one of 10 core epidemiology topics (Table 1). Next, students completed short online graded assessments of their understanding of the new concepts presented in these media based on the Just-in-Time Teaching (JiTT) pedagogy of Novak, Petterson, Gavrin, and Christian [15]. Because these JiTT assessments are taken in the interval between the student’s completion of at-home assignments (e.g., readings and/or videos) and the subsequent classroom meeting, they provide real-time feedback to the instructor about any remaining knowledge gaps, allowing him/her to make last-minute adjustments to the upcoming lesson plan. Originally developed in collaboration with the US Air Force Academy as a way to improve student preparedness and promote the use of class time for more active learning, JiTT has been associated with improved study habits, measurable cognitive gains, and increased academic performance in a variety of university-level Science, Technology, Engineering, and Mathematics (STEM) classes [16]. Our JiTT exercises consisted of a combination of essay, short-answer, and quantitative questions designed not only to test students’ integration of the material and ability to translate and apply their knowledge a new context, but also to identify weaknesses in understanding that needed to be addressed in the following discussion sections. Examples of two JiTT exercises are provided in Table 2. To standardize grading across discussion section leaders, JiTT exercises were graded according to a pre-specified rubric that evaluated student achievement on a 1–4 point scale across four competencies identified as key learning objectives of the course: application of epidemiologic concepts, application of epidemiologic methods, analytic thinking, and innovative thinking (Additional file 1: Table S1). At the end of each JiTT exercise, students were asked, “Is there anything that remains unclear from the lecture or readings on [topic] that you would like to discuss further in discussion section this week?” This served to highlight for section leaders areas of remaining confusion, as well as to encourage questions from students who might feel too shy or intimidated to admit uncertainty in front of their peers. Section leaders met weekly prior to the in-person discussion sections to review the JiTT exercise results and discuss alternative approaches to presenting material that students had not fully absorbed from the readings and lectures.

**Table 1:** Overview of Principles of Epidemiology

Lecture	Core Epidemiology Topic
Lecture 1	Introduction and History of Epidemiology
Lecture 2	Descriptive Epidemiology
Lecture 3	Infectious Disease Epidemiology
Lecture 4	Causal Inference
Lecture 5	Study Design I: Randomized Controlled Trials
Lecture 6	Study Design II: Cohort and Case-Control Studies
Lecture 7	Non-Comparability I: Random Error and Confounding
Lecture 8	Non-Comparability II: Selection Bias and Misclassification
Lecture 9	Screening
Lecture 10	Interaction and Generalizability

**Table 2:** Description of two Just-in-Time Teaching (JiTT) exercises

Example 1	
Topic	Cohort and Case-Control Studies
Activity	Use the following information to answer Questions 1–3 below. An article from the *New York Times* with the headline “Prevention: Fish Helps Reduce Risk of Polyps in Women” stated in the first sentence that “A new study has found that consumption of omega-3 fatty acids from fish is associated with a reduced risk for one kind of colon polyp, but only in women.”
Questions	1. How would you design a cohort study to evaluate the association between consumption of omega-3 fatty acids and colon polyps? What are some of the strengths and limitations of your cohort study? Please limit your answer to no more than one paragraph. 2. How would you design a case-control study to evaluate the association between consumption of omega-3 fatty acids and colon polyps? What are some of the strengths and limitations of your case control study? Please limit your answer to no more than one paragraph. 3. Which study design is better for answering this question? 4. Epidemiologic case-control studies often report increased risk of an event given exposure, but we know that we can only calculate the odds ratio in a case-control study as opposed to a risk ratio. Is it important to distinguish between a risk ratio and an odds ratio? When does the odds ratio approximate the risk ratio? When does it approximate the rate ratio? 5. Is there anything that remains unclear from the lecture or readings on cohort and case-control studies that you would like to discuss further in discussion section this week?
Example 2	
Topic	Random Error and Confounding
Activity	Students are asked to read a *New York Times* article, “Barnyard Dust Offers a Clue to Stopping Asthma in Children,” that describes a recent study comparing asthma prevalence among Amish and Hutterite children (https://www.nytimes.com/2016/08/04/health/dust-asthma-children.html?_r=0).
Questions	1. How does the design of this study deal with the issue of noncomparability that has plagued prior studies that have tried to contribute evidence to support the Hygiene Hypothesis? What are the potential sources of noncomparability that the authors have circumvented? Are there others that you think they might still be concerned about? Casting your mind back to the module on causal inference, which of Hill’s guidelines are met with the publication of this new study? Do you feel the weight of evidence is now sufficient to prove the Hygiene Hypothesis? Please limit your response to between one paragraph and one page, double spaced. 2. You are interested in calculating the population average BMI at the Mailman School of Public Health. Would you expect to have a wider or narrower confidence interval if you sampled 25 people vs. if you sampled 200 people? a. Wider b. Narrower c. There is not enough information to answer this question. 3. In a study of the possible effect of smoking on weight loss, you are concerned about potential confounding by alcohol consumption, which is positively associated with smoking and negatively associated with weight loss. What is one method you CANNOT use to control for confounding in either the design or analysis phase of this study? a. Stratification b. Regression c. Randomization d. Restriction e. Matching 4. In 2–3 sentences, please explain why you chose this response. 5. Is there anything that remains unclear from the lecture or readings on random error and confounding that you would like to discuss further in discussion section this week?

#### In class

Students then attended 2-h in-person discussion sections led by doctoral students in Epidemiology. In these sessions, section leaders spent the first 15–20 min reviewing course logistics (e.g., upcoming assignments and examinations) and clarifying outstanding questions, including both those posed by the students based on the week’s assigned reading and lecture, as well as those identified by the section leaders via the JiTT exercises; the next 45–60 min facilitating work in groups of 4–5 students on prepared exercises designed to apply the concepts covered in that week’s course materials; and the last 30–50 min leading full-class discussion. During this final segment, class time was allotted for supervised group project work, described below.

#### Additional elements maintained from the traditional format

1) In addition to their weekly work, students were assigned a semester-long group project in which they contextualized, analyzed, and reviewed a published scientific journal article describing an epidemiologic study related to a current public health problem. Students were responsible for meeting interim deadlines, and at the end of the semester each group delivered a Canvas web page and presented their findings orally during the final discussion section. 2) Section leaders each held a weekly drop-in office hour and were available to answer student questions by email throughout the semester. 3) Students were evaluated at midterm and at the end of the semester with 3-h in-class examinations.

### Evaluation and analysis

We examined students’ learning experiences and perceptions of the flipped classroom model using mixed methods. First, we compared the 2016 flipped classroom model to the 2015 traditional model. This was done by comparing characteristics of the students, midterm/final examination scores, and standard end-of-course evaluations, including open-ended student comments, across the two cohorts.

Next, we evaluated data on the flipped classroom model collected during the course of the Fall 2016 semester in four different domains. First, to measure changes in students’ self-reported knowledge, we administered pre- and post-course surveys using a 29-item version of the Student Assessment of their Learning Gains (SALG) instrument modified to match the learning objectives of our course [17]. The SALG evaluates five domains that influence student learning: teaching methods and class activities that facilitate learning, gains in topic-related knowledge, gains in skills, students’ overall enthusiasm for the subject, and improvements in students’ ability to integrate information. Second, it provided an opportunity for students to provide open-ended feedback on the strengths and weaknesses of the course, including comments about the flipped classroom model. Third, each section leader completed the Carl Wieman Science Education Initiative (CWSEI) Teaching Practices Inventory, a questionnaire designed to assess how a teacher prepares for and runs a science or mathematics course across eight domains, including what course information and supporting materials the teacher provides to students; how the teacher conducts the sessions; how the teacher handles assignments, feedback, grading, and course evaluation; what training the teacher receives; and whether the teacher has opportunities to collaborate with colleagues [18].

Finally, trained staff from Columbia’s Center for Teaching and Learning (CTL) conducted classroom observations using the Classroom Observation Protocol for Undergraduate STEM (COPUS) instrument, which characterizes how faculty and students spend their time in the classroom [19]. In successive 2-min intervals over the course of the class, the observer assesses what students and instructor are doing according to predetermined categories. Options for students include “Listening to instructor/taking notes, etc.,” “Working in groups on worksheet activity,” “Student asks question,” and “Presentation by student(s),” while options for instructors include “Lecturing,” “Listening to and answering student questions with entire class listening,” “Moving through class guiding ongoing student work during active learning task,” and “Administration.”

Data collection was completed by January 2017. Statistical analyses, including t-tests and chi-square tests where appropriate, were conducted using SAS version 9.4 (Cary, NC). Students and instructors provided consent for appearing in recordings in videos.

### Ethics and consent

Ethical approval to use de-identified existing data collected during the courses was obtained from the Institutional Review Board of Columbia University Medical Center (New York, NY).

## Results

### Student characteristics

In 2015 (traditional class model), a total of 72 students were enrolled in the course, including 59.7% (43) from the Department of Biostatistics, 15.3% (11) from the Department of Epidemiology, 8.3% (6) from the Department of Health Policy and Management, and 16.7% (12) from other departments. In 2016 (flipped classroom model), a total of 78 students were enrolled in the course, including 80.8% (63) from the Department of Biostatistics, 14.1% (11) students from the Department of Epidemiology, and 5.1% (4) from other departments. The composition of the student body enrolled in the course changed significantly from 2015 to 2016 (*p* < 0.01) due to changes in the programs the students were enrolled in, with a greater percentage from Biostatistics and smaller percentage from other departments in 2016 compared to 2015. Of note, in 2016, no students were enrolled from the Health Policy & Management department, as the course was no longer required for that degree.

### Data comparing 2015 to 2016

At the end of the Fall 2015 semester, 86.1% (62 out of 72) students in the traditional classroom model submitted course evaluations. At the end of the Fall 2016 semester, 80.8% (63 out of 78) students in the flipped classroom model submitted course evaluations.

There were no statistically significant differences in students’ assessment of the course across nine questions shared by the two evaluations (Table 3). For example, the majority of those who responded in 2015 and 2016 felt the time required per credit was “about the same” as other courses (62.9% (39) and 68.3% (43), respectively). Comparable percentages agreed or strongly agreed that they would recommend the course to others (79.0% (49) and 79.4% (50)), that the discussion sections supported the learning goals of the course (91.9% (57) and 87.3% (55)), and that they felt well or very well prepared to demonstrate the competencies of the course (80.6% (50) and 84.1% (53)).

**Table 3:** Course evaluations from Principles of Epidemiology, Fall 2015 vs. 2016

Component	2015 *N* = 62	2016 *N* = 63	*P*-value
Found the time required per credit for this course compared to other courses is about the same	62.9% (39)	68.3% (43)	0.53
Agreed or strongly agreed the requirements of the course were reasonable for the course credits allotted	83.9% (52)	88.9% (56)	0.41
Agreed or strongly agreed the discussion sessions supported the learning goals of the course.	91.9% (57)	87.3% (55)	0.40
Agreed or strongly agreed they would recommend the course to other students	79.0% (49)	79.4% (50)	0.96
Agreed or strongly agreed the course contributed to the pursuit of their professional goals.	82.3% (51)	85.7% (54)	0.60
Felt well or very well prepared to demonstrate the competencies of the course	80.6% (50)	84.1% (53)	0.61
Felt well or very well prepared to apply course concepts and skills to solve public health problems	87.1% (54)	81.0% (51)	0.35

Course midterm and final examination scores are provided in Table 4. There was no significant change in mean ± SD midterm (91.4 ± 9.3 vs. 93.4 ± 6.1) or final examination (90.5 ± 9.4 vs. 91.2 ± 6.9) scores from 2015 to 2016. Results were not significantly different when limited to students only from the Departments of Biostatistics and Epidemiology (midterm: 91.9 ± 9.1 vs. 92.0 ± 12.5; final: 91.9 ± 8.5 vs. 90.1 ± 12.5).

**Table 4:** Midterm and exam results from Principles of Epidemiology, Fall 2015 vs. 2016

	N	Median	Mean	Std Dev	Range	*P*-value^a^
Midterm 2015	71	94.0	91.4	9.32	50.5–100	0.152
Midterm 2016	78	94.4	93.4	6.05	74.5–100	
Final 2015	71	94.0	90.5	9.36	59.5–100	0.618
Final 2016	78	92.5	91.2	6.90	68.8–100	

^a^Mean difference from 2015 to 2016

### Data collected in fall 2016

Additional file 1: Table S2 provides select items from pre- and post-course SALG surveys used to measure changes in students’ self-reported knowledge across the Fall 2016 semester. Of the 78 course enrollees, 87.2% (68) answered the pre-course survey and 61.5% (48) answered the post-course survey. For all components, the mean score of self-reported knowledge increased from pre-course to post-course.

Questions were added to the end-of-course evaluation in 2016 that specifically addressed the flipped classroom model (Additional file 1: Table S3). In 2016, 57.1% (36) of respondents found watching video lectures at home to have a somewhat or strongly positive impact on their time management while 27.0% (17) found it to have a somewhat or strongly negative impact. Approximately equal percentages found it easier or harder to absorb material via recorded compared to live lectures (36.5% (23) vs. 33.3% (21)). Most of the students who responded agreed or strongly agreed that the weekly JiTT exercises provided a helpful review of the readings and lectures (74.6% (47)) and the group project supported the learning goals of the course (73.0% (46)).

The end-of-course evaluations included free-text sections in which students were invited to comment on the strengths of the course and areas for improvement. Of the 63 students who submitted feedback for course evaluations in Fall 2016, 74.6% (47) provided comments that fell into one of these two categories (60.3% of the total course enrollment). Selected results from the qualitative surveys are provided in Additional file 1: Table S4. Students identified the flexibility offered by the pre-recorded lectures, the intermediate deadlines to complete discussion section preparation materials, and the ability of section leaders to further explain and clarify concepts in person as key strengths of the course, while their suggestions for further improvement focused on ways to increase regular interaction with lecturers. Additional file 1: Table S5 presents a summary of themes that were mentioned by more than three students.

All six discussion section leaders filled out the CWSEI Teaching Practices Inventory. Three (50%) reported lecturing for 0–20% of a typical section, while three (50%) reported lecturing for 20–40% of a typical section. Three (50%) section leaders reported spending 0–10% of a typical section discussing the process by which a theory/model/concept was developed, while two (33.3%) reported spending 11–25% and one (16.7%) reported spending > 25% of their time on this aspect. Four (66.7%) of the section leaders discussed how to teach the course with their colleague(s) very frequently, while 2 (33.3%) did not.

Additional file 1: Figure S1 shows the breakdown of time spent on various activities by the students and section leaders according to observations conducted using the COPUS instrument. Students spent approximately half (48.9%) of the discussion section time working on group activities, 26.7% listening, 11.1% asking questions, and 13.3% answering questions. Section leaders spent most of their time observing student/group activities (18.3%), lecturing (16.7%), and guiding the class through an activity (15%).

## Discussion

Objective measures of student performance in our introductory epidemiology class and subjective course evaluations were similar when we compared the flipped classroom model offered in 2016 to the traditional model offered in 2015. We observed no statistically significant differences in midterm or final examination scores between the 2 years, in keeping with the University of Saskatchewan study [14] and a study of public health students participating in a flipped Environmental and Occupational Health course in which no difference was reported in mean examination scores [12]. By contrast, a study of a flipped first-year course on pharmaceutics reported a slight increase in final exam scores [10]. We also did not observe differences in end-of-year student assessments between the traditional and flipped models.

Compared to attending scheduled in-person lectures, 57.1% (36) of those responding to the end-of-course evaluation in 2016 found watching lectures at home to have a somewhat or strongly positive impact on their time management while 27.0% (17) found it to have a somewhat or strongly negative impact. Student evaluations included both positive and negative open-ended comments. Overall, the positive comments reflected our desired goals for implementing a flipped classroom, including that the JiTT exercises held students accountable for their own learning, the sections helped to clarify targeted issues, the group project improved collaboration and peer learning, and the online lectures increased flexibility and convenience for busy students. Flexibility is an important component of the flipped classroom model that has been highlighted in other studies [12, 14]. The negative comments highlighted some of the challenges of a flipped classroom, particularly loss of real-time interaction with lecturers and the perception of the model as a cost-cutting maneuver.

This study has a number of important strengths. The mixed methods used to evaluate this flipped classroom intervention provide a multifaceted view of its successes and areas for improvements. We were able to collect both quantitative and qualitative data from students, including their responses to the end-of-course evaluations, the pre- and post-course SALG questionnaires, and their midterm and final examination grades. In addition, we gathered subjective data from discussion section leaders via their CWSEI Teaching Practices Inventories as well as objective data about how they ran their classrooms from the COPUS instrument administered by trained observers. Other strengths include the consistency of our teaching staff (four of the six section leaders taught in both years) and our ability to draw on the expertise of Columbia’s CTL staff for advice on designing and administering our evaluation, including the selection of validated tools and methods.

There are two main limitations of this evaluation. First, the composition of the student body enrolled in the course changed between years, and in 2016, we had no students from the Health Policy & Management department, as our course was no longer required for that degree. Students from different departments often have different skill sets, learning styles, and academic goals, which may influence their preference for a traditional vs. flipped classroom model. When we limited our analyses of exam scores to only those in the Departments of Biostatistics and Epidemiology, we did not observe any differences. Second, we had incomplete participation in our online assessments. The response rates for the end-of-course evaluations were comparable between 2015 and 2016 (86.1% vs. 80.8%). In 2016, however, twenty fewer students completed the SALG questionnaire at the end of the semester than had completed the questionnaire at the beginning, leaving open the possibility that selection bias may have influenced the results of our pre-post comparison during the year the flipped classroom was implemented. An additional minor limitation is that approximately 25% of the exam questions were changed between 2015 and 2016, as has been standard practice from year to year in teaching this course. We have no reason to believe that these changes would significantly influence student performance metrics. Finally, we did not assess students’ media literacy at the beginning of the 2016 semester, which would have been helpful to analyze whether this facility is an important factor in determining which students benefit most from the flipped classroom approach.

## Conclusions

There was no significant difference in students’ performance on quantitative assessments comparing the traditional format to the flipped classroom format. The flipped format did allow for flexibility and greater applied learning opportunities at home and during in-class discussion sections. In response to feedback from students, section leaders, and classroom observers, we have identified four areas in which to make changes to improve the flipped classroom experience in the future. First, to address students’ desire to ask questions during lectures, we are now exploring the use of interactive software such as VoiceThread, a tool designed for online courses that allows for asynchronous communication through text, voice recording, and video or image upload [20]. Students will be able to annotate lecture slides with comments that will be visible both to section leaders, who will be able to respond within a short amount of time, and to other students, who may want to contribute to the discussion. Second, to increase the value of the recorded material, we will create a searchable index of the lectures to facilitate students’ ability to review key terms and concepts. Third, to improve consistency of teaching across discussion sections, in addition to our pre-semester section leader training, we are establishing an ongoing monitoring system in which faculty and staff from the CTL will conduct regular observations of discussion sections. Based on their assessment, additional training will be administered as needed. Finally, we will adjust the group project assignment to foster more consistent engagement among students with different backgrounds.

## Additional file

Additional file 1 : **Figure S1** Classroom observation conducted using the Classroom Observation Protocol for Undergraduate STEM (COPUS) instrument. **Table S1** Grading Rubric for JiTT exercises. **Table S2** Comparison of pre- and post-course self-perceived knowledge from the modified Student Assessment of their Learning Gains survey. **Table S3** Additional questions added to the end-of-course evaluation in 2016 that specifically addressed the flipped classroom model. **Table S4** Themes from qualitative evaluation of student feedback in the final course evaluation, Principles of Epidemiology, Fall 2016. **Table S5** Selected summary of qualitative feedback on the strengths and areas for further improvement in the final course evaluation, Principles of Epidemiology, Fall 2016. (DOCX 86 kb)

## Abbreviations

CWSEI: Carl Wieman Science Education Initiative
CTL: Center for Teaching and Learning
COPUS: Classroom observation protocol for undergraduate STEM
JiTT: Just-in-Time-Teaching
MSPH: Mailman School of Public Health
MS: Master of science
STEM: Science, technology, engineering, and mathematics
SALG: Student assessment of their learning gains
