# Supplementary material for: Evaluation of a flipped classroom approach to learning introductory epidemiology
Source: BMC Med Educ. 2018 Apr 2;18:63. doi: 10.1186/s12909-018-1150-1 (PMC5879803; doi:10.1186/s12909-018-1150-1)
Supplement: Supplementary file 1 — : Figure S1 Classroom observation conducted using the Classroom Observation Protocol for Undergraduate STEM (COPUS) instrument. Table S1 Grading Rubric for JiTT exercises. Table S2 Comparison of pre- and post-course self-perceived knowledge from the modified Student Assessment of their Learning Gains survey. Table S3 Additional questions added to the end-of-course evaluation in 2016 that specifically addressed the flipped classroom model. Table S4 Themes from qualitative evaluation of student feedback in the final course evaluation, Principles of Epidemiology, Fall 2016. Table S5 Selected summary of qualitative feedback on the strengths and areas for further improvement in the final course evaluation, Principles of Epidemiology, Fall 2016. (DOCX 86 kb) [file 12909_2018_1150_MOESM1_ESM.docx]

**Additional file 1**

**Figure S1: Classroom observation conducted using the Classroom Observation Protocol for Undergraduate STEM (COPUS) instrument**

What students are doing: What discussion section leader is doing:


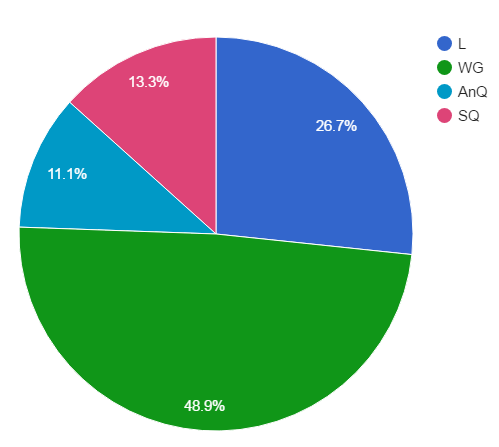

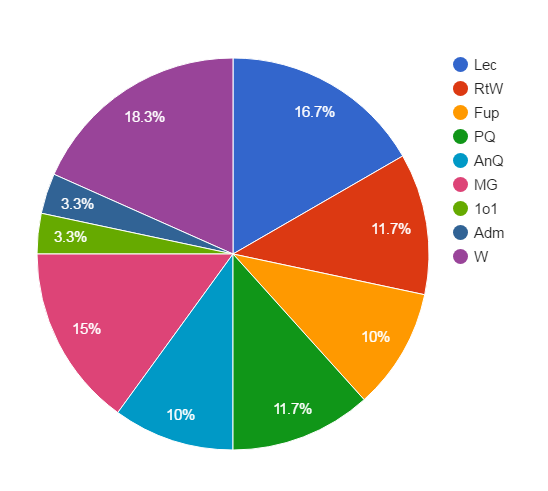


**Key:**

L = listening to discussion section leader/taking notes, etc.

WG = Working in groups on worksheet activity

AnQ = Student answering a question posted by the instructor with rest of class listening

SQ = Student asking a question

Lec = Lecturing/presenting content

RtW = Real-time writing on board

FUp = Follow-up/feedback on activity to entire class

PQ = Posing question to students

AnQ = Listening to and answering student questions with entire class listening

MG = Moving through class guiding ongoing student work during active learning task

1o1 = One-on-one extended discussion with one or a few individuals, not paying attention to the rest of the class

Adm = Administration (assigning homework, returning tests, etc.)

W = Observing/listening to student or group activities

**Table S1: Grading Rubric for JiTT exercises**

| **Competency** | **Question Type** | **Needs work (1)** | **Satisfactory (2)** | **Proficient (3)** | **Advanced (4)** | **Score** |
| --- | --- | --- | --- | --- | --- | --- |
| **Application of Epidemiologic**  **Concepts** | Any definition or interpretation questions | Lack of clarity in defining and in interpreting epidemiologic concepts | Lack of clarity in defining but attempts to interpret epidemiologic concepts with some minor misinterpretations | Clearly defines and interprets epidemiologic concepts | Clearly defines and interprets epidemiologic concepts in an advanced way |  |
| **Application of Epidemiologic Methods** | Any calculation and short answer questions | Did not take the right approach for calculations and short answer questions | Took the right approach for calculations and short answer questions but interpretation is unfounded/error(s) in calculations | Took the right approach for calculations and short answer questions and interpretation is basic | Took the right approach for calculations and short answer questions and interpretations are highly advanced |  |
| **Analytic Thinking** | Compare/  contrast concepts, deeper analysis | Does not compare or contrast complicated concepts and does not provide deeper analysis | Attempts to compare and contrast complicated concepts but does not provide deeper analysis | Compares and contrasts complicated concepts and provides limited deeper analysis | Compares and contrasts complicated concepts and provides deeper analysis in an advanced fashion |  |
| **Innovative**  **Thinking** | Application of epi concepts to new situations | Major mistakes in applying epidemiologic concepts to new scenarios | Minor mistakes in applying epidemiologic concepts to new scenarios, but insufficient analysis | Successfully applies epidemiologic concepts to new scenarios and provides adequate analysis | Successfully applies epidemiologic concepts to new scenarios and provides excellent analysis |  |

**Table S2: Comparison of pre- and post-course self-perceived knowledge from the modified Student Assessment of their Learning Gains survey**

| **Component** | **Mean score pre-course** | **Mean score post-course** |
| --- | --- | --- |
| Presently, I understand the relationships between epidemiology concepts | 3.56 | 4.44 |
| Presently, I understand the role of epidemiology in public health | 3.37 | 4.35 |
| Presently, I can formulate an epidemiologic research question | 2.52 | 3.67 |
| Presently, I can critique a published epidemiologic study | 2.51 | 3.92 |
| Presently, I can work effectively with others to prepare and present an oral presentation | 3.39 | 4.40 |
| Presently, I am enthusiastic about epidemiology | 3.71 | 4.11 |
| Presently, I am interested in taking or planning to take additional classes in epidemiology | 3.21 | 3.83 |
| Presently, I am confident that I understand the basics of epidemiology | 3.10 | 4.15 |
| Presently, I am confident that I can do well in this course or other epidemiology courses | 3.59 | 4.08 |

Note: Mean score was calculated, where 1=not at all, 2=just a little, 3=somewhat, 4=a lot, 5=a great deal.

**Table S3.** Additional questions added to the end-of-course evaluation in 2016 that specifically addressed the flipped classroom model

| **Question** | **Response options** |
| --- | --- |
| Some people find watching videos to be a more efficient use of their time than attending scheduled in-person lectures, while others find themselves falling into a habit of procrastination. Do you feel watching videos had a positive or negative impact on your time management? | Strongly Negative  Somewhat Negative  Neither Positive nor Negative  Somewhat Positive  Strongly Positive |
| I find it _________ to absorb the material via the video lectures compared to live lectures. | A Lot Easier  Somewhat Easier  Neither Easier Nor Harder  Somewhat Harder  A Lot Harder |
| I found the weekly assignments to be a helpful review of the readings and lectures. | Strongly Disagree  Disagree  Neutral  Agree  Strongly Agree |
| The group project supported the learning goals of the course. | Strongly Disagree  Disagree  Neutral  Agree  Strongly Agree |

**Table S4.** Themes from qualitative evaluation of student feedback in the final course evaluation, Principles of Epidemiology, Fall 2016

|  | **Described as a strength** | **Described as an area for further improvement** |
| --- | --- | --- |
| **Theme** | (N=25) | (N=22) |
| **Course content** | 7 | 2 |
| **Course format** | 7 | 11 |
| **Group project** | 3 | 3 |
| **Effectiveness of discussion section leaders** | 7 | 2 |

**Table S5.** Selected summary of qualitative feedback on the strengths and areas for further improvement in the final course evaluation, Principles of Epidemiology, Fall 2016.

| Strengths | |
| --- | --- |
| Course content | Course content was relevant and useful, and instruction was effective and helpful. |
| Course format | It's efficient for us to learn it by video lectures, because it's convenient for us to manage our time. |
|  | I liked the online lecture model because I could refer back to it for review*.* |
|  | I found this course to be convenient in that we could watch the lecture videos at home, and having the quiz [deadlines] forced me to watch the lecture video before then. |
| Effectiveness of discussion section leaders | My discussion section leader was friendly and gave excellent help outside of class. |
|  | Discussion section leaders effectively managed the section time and provided thoughtful commentaries and succinct summaries of concepts in and outside of class. |
| Areas for further improvement | |
| Course format | I liked and disliked the video format. On one hand, it was nice to be able to watch it whenever. On the other hand, you can't ask questions, you don't get to interact with faculty, and your learning is limited to the slides. I would have preferred to have in-person lectures that were also video-recorded. That way, I can go to class and re-watch the lectures later when I was studying. |
